# Let There be Light! Light as an Engine and Regulator in Synthetic Cells

**DOI:** 10.1002/anie.4174230

**Published:** 2026-05-06

**Authors:** Matthew E. Allen, Saskia Frank, Mehaiarii Louis, Atreyee Saha, Ali Heidari, Seraphine V. Wegner

**Affiliations:** ^1^ Institute of Physiological Chemistry and Pathobiochemistry University of Münster Münster Germany

**Keywords:** light, photoswitchable, spatiotemporal regulation, synthetic biology, synthetic cells

## Abstract

Synthetic cells, assembled from defined molecular components, are designed to mimic the features, form, and function of living cells. Light has emerged as a uniquely precise, biorthogonal, and non‐invasive stimulus for regulating and energizing these systems, enabling chemical inhomogeneity and an out‐of‐equilibrium state central to many cellular processes. This review highlights the biological behaviors and functions that light has helped recreate in synthetic cells, including compartmentalization, energy supply and metabolism, protein synthesis, communication, growth, shape change and division, and motility. We survey the breadth of light‐responsive components incorporated into synthetic cells, spanning photoswitchable and photocleavable small molecules, photoswitchable proteins, photocatalysts, nanoparticles, and photosynthetic organelles or organisms. Finally, we offer a perspective on key design considerations such as wavelength, reversibility, integration, biocompatibility, multicolor regulation, and biohybrid strategies. Together, these advances chart promising routes toward more dynamic, energy‐autonomous, and programmable synthetic cells that will deepen our understanding of cellular functions and enable emerging biotechnological applications.

## Graphical abstract

Cells are out‐of‐equilibrium systems that regulate their spatiotemporal features. In biomimetic synthetic cells, light offers a unique way for energizing and regulating events in space and time. This review explores the implementation of light‐responsive tools in synthetic cells to replicate biological functions and enable new bioinspired behaviors with promising biotechnological applications.

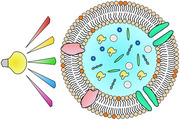

## 1 Introductions

Synthetic cells (also known as artificial cells and protocells) are engineered, cell‐sized platforms designed to mimic living cells in both form and function [1]. Using this biomimicry, synthetic cells aim to deepen our understanding of cellular behavior [2], shed light on the emergence of life, and provide versatile bioinspired chassis for biotechnological applications [3]. Synthetic cells are constructed using either a top‐down or bottom‐up approach. The top‐down approach involves deconstructing living organisms to define a minimal set of genes essential for life [4], whereas the bottom‐up approach (focused on in this review) creates cell‐like systems by assembling diverse molecular building blocks [5]. By utilizing molecularly defined biological and synthetic components, synthetic cells can replicate essential cellular functions. Through the integration of different functional modules, the ultimate goal is to generate systems that increasingly resemble living systems in their behavior and complexity [6]. Achieving these life‐like features often requires chemical heterogeneity, spatiotemporal regulation, and out‐of‐equilibrium conditions, which can be generated through chemical and non‐chemical inputs [7, 8].

Light has been a persistent environmental factor throughout the history of life on Earth. While it may not have been essential for the origin of life, it has been a major evolutionary driver, giving rise to the development of photoperception across the tree of life, including phototropism in plants [9], phototaxis in microorganisms, as well as vision and circadian clocks [10] in animals. Equally important, most life forms depend directly or indirectly on light as an energy source, captured through photosynthesis [11]. It is therefore unsurprising that light has become a central element in the design of synthetic cells, used both to regulate and to energize these systems. Light offers several advantages: it can be delivered with unparalleled spatial and temporal precision [12], be applied in a contact‐free manner [13], is highly tunable through exposure time and intensity [14], and offers the possibility to control multiple functions independently with different wavelengths of light [15]. These attributes make light an exceptionally attractive tool for synthetic cell research and biotechnological applications in general. Consequently, light has been extensively employed to control systems, ranging from synthetic materials [16] to living cells [17], using photocleavable and photoswitchable small molecules and proteins, as well as photocatalytic and photothermal processes [17]. This has generated a rich toolbox of light‐responsive components and optogenetic tools, which provide photoregulation in living cells and can be incorporated into synthetic cells to produce life‐like behaviors.

**FIGURE 1:**
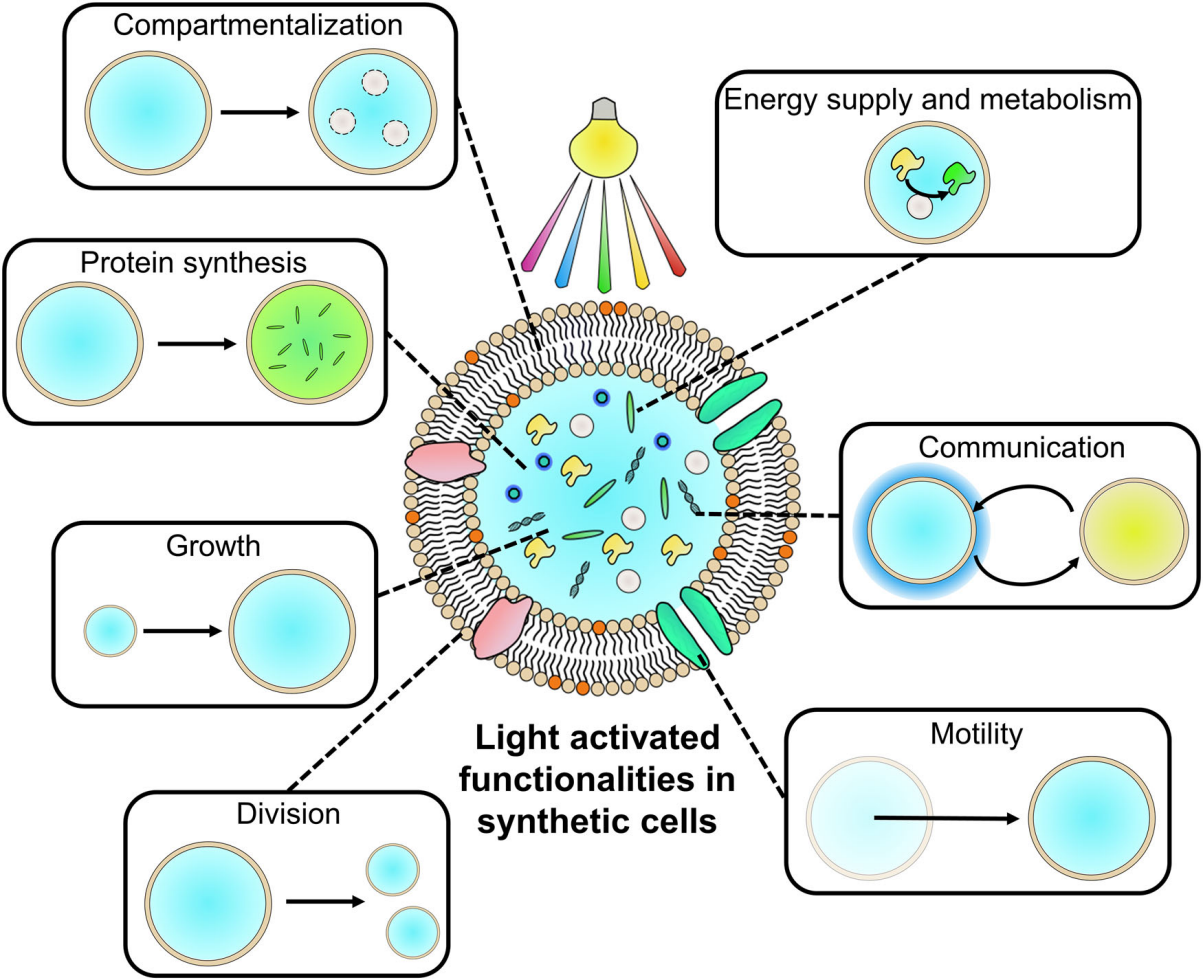
A schematic outlining key synthetic cell functionalities that can be induced by light. These include (from top to bottom): light‐triggered formation of internal compartments, light‐controlled internal energy supply and metabolism, light‐activated internal protein synthesis, light‐activated communication between different synthetic cell populations, light‐triggered growth of a synthetic cell from amphiphiles, light‐guided motility, and light‐induced division.

In this review, we will focus on how light can be used to elicit life‐like functionalities in synthetic cells. In particular, we will explore how light has enabled the replication of the key cellular functions, including compartmentalization, energy supply and metabolism, protein synthesis, communication, growth, shape changes and division, and motility (Figure 1), before we conclude with a perspective on how light‐responsive elements can be further exploited to advance the next generation of synthetic cells.

## 2 Light‐Enabled Functionalities in Synthetic Cells

### 2.1 Compartmentalization

Compartmentalization of content is widely seen as a prerequisite for life, as it establishes a physical and functional boundary between living systems and the external environment [18]. At the same time, compartmentalization occurs across multiple scales, from tissues composed of different cell types to a variety of organelles within the cell, with each maintaining a distinct chemical environment and performing discrete functions [19]. Light has played a central role in the compartmentalization of synthetic cells, ranging from remodeling compartments and prototissues [20] to modifying the properties of existing synthetic cells, generating protein patterns, and initiating the release from compartmentalized structures [21].

**FIGURE 2:**
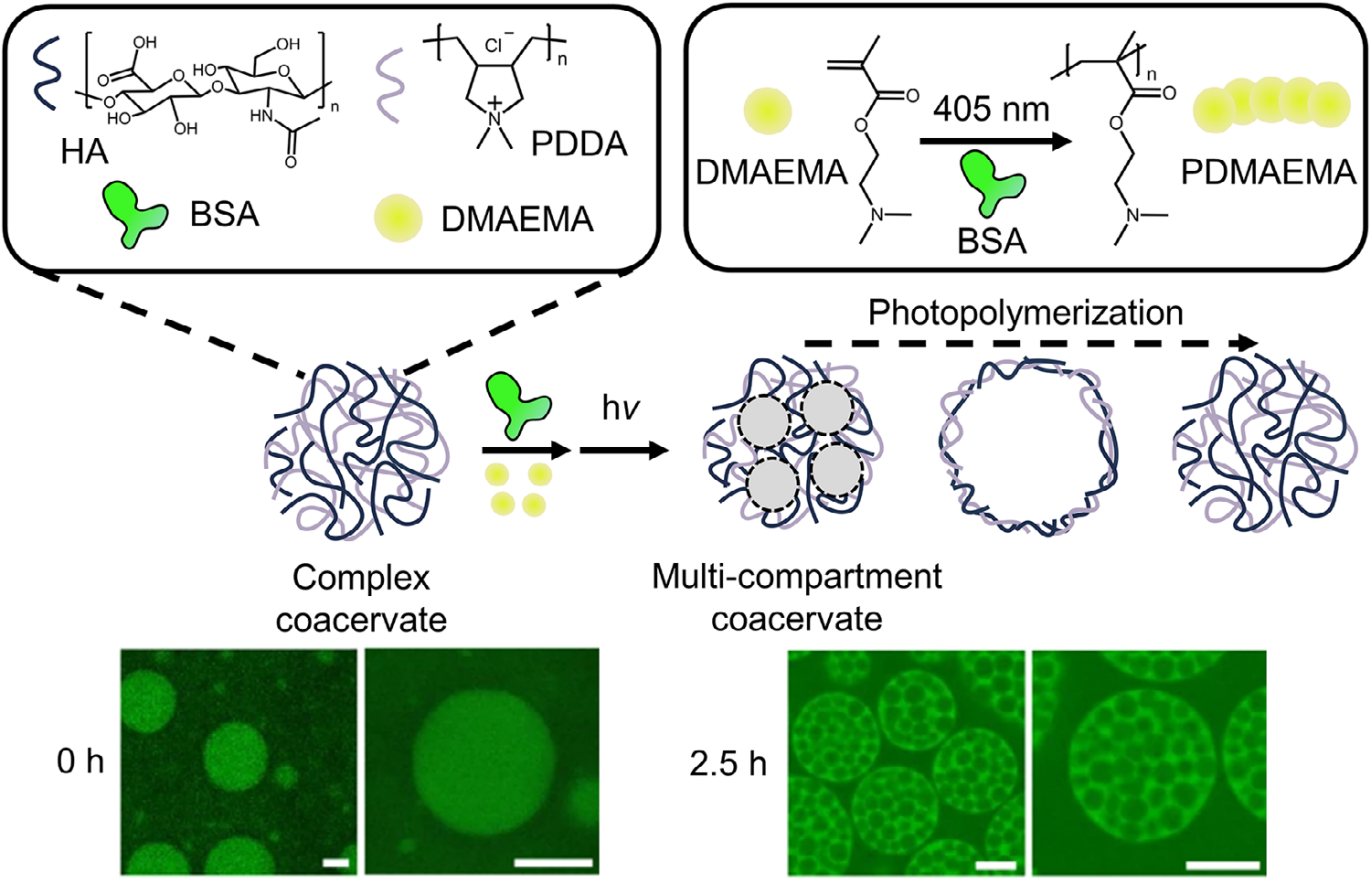
Light‐triggered compartmentalization in synthetic cells. A schematic depicting the assembly of multicompartment, coacervate‐based synthetic cells through the use of BSA mediated photopolymerization of DMAEMA. The fluorescence microscopy images show the synthetic cells after 0 and 2.5 h of photopolymerization and the evolution of the multicompartment structure. The scale bars are 5 µm. The figure is adapted with permission from ref [32], 2025 the authors CC‐BY 4.0.

Light has been widely used to create and sculpt compartmentalized structures from diverse biological and synthetic building blocks. For example, lipid membrane‐bound compartments have been formed from photoswitchable lipid‐like molecules that, upon illumination, generate giant vesicles and even multivesicular, tissue‐like structures [22]. Similar to the protein‐based membraneless organelles found in cells, synthetic membraneless organelles have been created within synthetic cells using the blue‐light‐responsive protein Cry2olig‐IDRs. Remarkably, these organelles were shown to recruit nitric oxide synthase to generate nitric oxide and enable therapeutic activity in melanoma models [23]. Similarly, DNA‐based organelles and structures were generated within synthetic cell systems through the light‐driven assembly of DNA condensates under UV light (302 nm [24], 320 nm [25], or 365 nm [26]) or through photopolymerizing DNA hydrogels of defined shapes and sizes under violet light (405 nm) [27]. More recently, UV light (320 nm) has been used to also assemble hybrid DNA‐RNA condensates within water droplets, which can incorporate streptavidin, locally recruiting biotinylated molecules [28]. Light has further been used to pattern proteins on lipid membranes and lipid‐based synthetic cells through the use of either the blue‐light‐switchable disordered tether to improved light‐induced dimer (disiLID) and nano protein pair [29], the red‐light‐responsive phytochrome B (PhyB) and phytochrome‐interacting factor (PIF) protein pair [30], or the blue‐light‐responsive improved light‐induced dimer (iLID) and SspB‐mScarlet‐I‐VCA protein pair, the latter inducing localized actin polymerization [31]. These approaches establish robust asymmetry and selective protein localization on membrane‐bound compartments. Beyond biomolecular systems, bovine serum albumin (BSA)‐mediated photopolymerization of methacrylic acid 2‐(dimethylamino) ethyl ester (DMAEMA) within coacervate‐based synthetic cells has led to the formation of multiple, highly stable internal compartments within coacervates [32] (Figure 2).

Aside from generating compartmentalized structures at the subcellular scale, light has also been used to trigger assembly and shape changes at the prototissue scale. Through utilizing orthogonal blue (480 nm) and red (630 nm) light‐switchable protein‐protein interactions for adhesions between cell‐sized polystyrene beads, assemblies with distinct self‐sorting patterns can be formed depending on the color of illumination [33]. Moreover, in a synthetic tissue composed of synthetic cells containing poly(ethylene glycol)‐stabilized gold nanoparticles and a thermoresponsive poly(N‐isopropylacrylamide) cortex, illumination with green light (520 nm) induced reversible contraction and shape changes. In this case, localized heating around the gold nanoparticles caused the contraction of the poly(N‐isopropylacrylamide) cortex, leading to overall shape changes [20].

Another important aspect regulated by light in synthetic cells is the permeability of compartments. Lipid vesicle organelles containing photopolymerizable lipids have been used to release small molecule cargo (fluorescein di‐β‐*D*‐galactopyranoside, 657 Da) into the lumen of synthetic cells under UV light (254 nm), where the released cargo could initiate an enzymatic reaction confined within the lumen [34]. In another example, synthetic cells containing organelles of photopolymerizable lipids released Tobacco Etch Virus (TEV) protease (27 kDa) under UV light, enabling the removal of a bulky protease recognition domain attached to connexin‐43 in the synthetic cell lumen. This triggered light‐mediated nanopore formation and the subsequent release of molecules from the synthetic cells [21]. In a similar manner, synthetic cells containing organelles of photopolymerizable lipids have used the release of TEV protease to unbind cargo bound to actin cytoskeletons within the synthetic cells [35]. In another approach, light has been used to build up chemical gradients in synthetic cells to power transport across membranes. For example, photocaged adenosine triphosphate (ATP) in proteo‐giant unilamellar vesicles (GUVs) has been used to activate ATP‐binding cassette (ABC) transporters, resulting in unidirectional substrate (labeled peptide ∼1.7 kDa) transport against steep concentration gradients [36]. Finally, synthetic microcapsules housing a photoswitchable catalyst can, under blue light, generate a chemical gradient that draws external objects into the microcapsule through a micropore, which can be up to 1.35 µm in diameter. Using this strategy, the capsules have captured bacteria and nanoparticles, creating a cell mimic with internal compartments [37].

### 2.2 Energy Supply and Metabolism

A constant supply of energy and the associated metabolic processes enable cells to maintain their out‐of‐equilibrium state and synthesize all essential components required for self‐sustenance. ATP, the most common chemical energy carrier in nature, provides the chemical driving force for continual cellular function and integrity, including anabolic biosynthetic reactions, cell motility and signal transduction [38]. Ultimately, most of this chemical energy originates from photosynthesis, which converts light energy into chemical energy, including in the form of ATP. Analogously, the conversion of light energy to chemical energy to power metabolism constitutes an important focus in synthetic cells [39].

**FIGURE 3:**
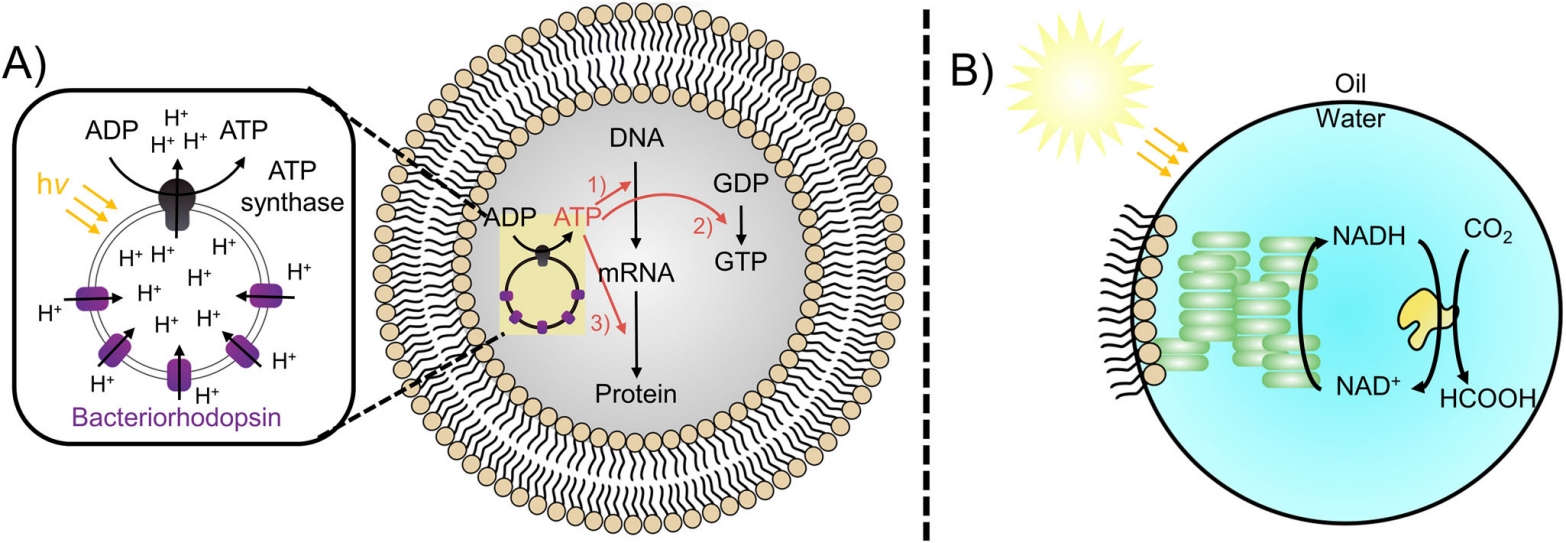
Energy supply through light in synthetic cells. (A) Schematic of a synthetic cell with a photosynthetic organelle containing bacteriorhodopsin and ATP synthase. The ATP produced upon light illumination supports transcription of mRNA (1), guanosine 5’‐triphosphate (GTP) synthesis (2), and translation (3). (B) An illustration of a synthetic cell module that converts CO_2_ into formate (HCOOH) under light irradiation. The module consists of a thylakoid membrane interfaced with CdTe quantum dots, integrated with enzymatic cofactors and encapsulated within water‐in‐oil droplets. Part (A) is adapted with permission from ref [41], 2019 the authors CC‐BY 4.0. Part (B) is adapted with permission from ref [49], 2023 the authors CC‐BY 4.0.

By far the most prevalent method for producing ATP in synthetic cells relies on combining light‐induced proton gradients with ATP synthase. For example, bacteriorhodopsin (bR), a light‐driven proton pump, generates a proton motive force across the lipid membrane of a synthetic cell, which ATP synthase subsequently exploits to produce ATP [40]. Building on this energy module, bR and ATP synthase have been co‐embedded within organelles inside a synthetic cell equipped with a cell free expression (CFE) system. Here, the light‐generated ATP supported transcription, translation and GTP synthesis, driving the formation of additional artificial organelle components and establishing a positive feedback loop that enhanced light driven ATP production, thereby paving the way toward energetically autonomous synthetic cells [41] (Figure 3A). In another example, this energy module was integrated into a synthetic cell equipped with a blue light‐activated adhesion module, allowing the synthetic cell to adhere in environments that support its ATP synthesis [42]. To accelerate ATP production, the bR of *Halobacterium salinarum* was incorporated into a synthetic cell built out of plasmonic colloidal capsules, where the plasmonic resonance accelerated the light‐induced proton pumping kinetics of bR, consequently increasing ATP generation by ATP synthase in a second population of synthetic cells [43].

An alternative route for generating proton gradients in synthetic cells involves components of the photosynthetic machinery, such as photosystem II (PSII), which produces protons through the photooxidation of water [44]. The ATP generated in this way was used to power enzymatic carbon fixation, yielding acetyl‐CoA, a key intermediate in anabolic metabolism, and oxalo‐acetic acid, thereby mimicking photosynthesis in synthetic cells [45]. In this system, the addition of the pigment protein phycocyanin enhanced PSII's light‐harvesting efficiency via energy transfer, significantly improving photosynthetic performance [45]. Meanwhile, switchable light‐harvesting organelles incorporating two different photoconverters, PSII and bacteria‐derived proteorhodopsin (PR), along with ATP synthase have also been developed. When encapsulated in synthetic cells, red light activated PSII stimulated ATP synthesis, while green light suppressed ATP synthesis by abolishing the proton gradient through PR activity. Consequently, this dual energy module enabled light‐controlled regulation of two ATP‐dependent processes, carbon fixation and actin polymerization [46].

Besides using light‐harvesting proteins, hybrid systems that combine synthetic cells with natural photosynthetic organisms or organelles have been employed for light‐dependent ATP generation and metabolism. For example, synthetic cells with trapped bacterial chromatophores from *Rhodobacter sphaeroides*, which contain the full machinery for light‐driven proton gradients and ATP synthesis, produced ATP sufficient to drive DNA transcription [47]. Similarly, encapsulating photosynthetic organisms such as cyanobacteria within synthetic cells enabled glucose production via CO_2_ fixation, which subsequently powered enzymatic cascade reactions starting from glucose [48]. Thylakoid membranes isolated frm spinach chloroplasts have also been placed into water‐in‐oil microdroplets to create photosynthetic microcompartments capable of CO_2_ fixation and operating a complete metabolic cycle of 16 enzymes (crotonyl‐coenzyme A (CoA)/ethylmalonyl‐CoA/hydroxybutyryl‐CoA (CETCH) cycle) for continuous carbon fixation [11]. Furthermore, embedding CdTe quantum dots on the thylakoid membranes enhanced the supply of photogenerated electrons, and thus the regeneration of NADH, NADPH and ATP, as well as CO_2_ reduction to formate [49] (Figure 3B).

Beyond biological photosystems, fully synthetic photocatalysts can power metabolism in synthetic cells with light. In addition to photopolymerization reactions discussed earlier in regards to compartmentalization, photoredox catalysis provides an alternative means of light‐driven metabolism. For instance, the key redox cofactor nicotinamide adenine dinucleotide has been regenerated in synthetic cells via photocatalysis in both its reduced (NADH) [50] and oxidized (NAD^+^) forms [51]. Similarly, white light‐induced water oxidation has also been achieved in synthetic cells and prototissues using encapsulated ruthenium complexes [52] as well as white light‐mediated CO_2_ reduction through cobalt complexes embedded in lipid membranes [53]. Alternatively, coacervate organelles containing a photocatalyst within a synthetic cell degraded methylene blue under blue light irradiation [54]. Overall, photocatalytic chemistries provide a versatile toolkit for powering synthetic metabolism using light [55, 56] and substantial opportunities remain for expanding this approach.

### 2.3 Protein Synthesis

The synthesis of proteins, as functionally the most diverse class of biomolecules, is a significant feature of living cells, and the regulation of their synthesis is a key contributor to cellular adaptability. Accordingly, synthetic cells encapsulating CFE systems, based either on cellular extracts or on purified components, have been developed to replicate transcription/translation and to produce a broad range of proteins in vitro [57]. Despite the wide variety of light‐responsive strategies available for regulating protein synthesis at the level of transcription and translation via modified nucleic acids and optogenetic tools both in vitro and in vivo [58, 59], light has been utilized to photoregulate CFE systems inside synthetic cells only in a few examples.

**FIGURE 4:**
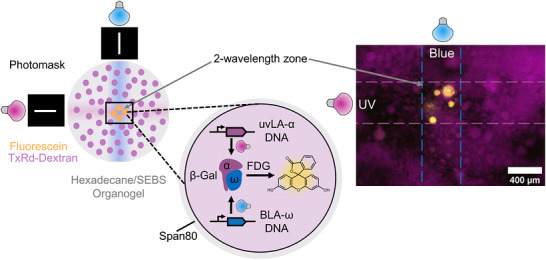
Light‐initiated CFE within synthetic cells. Schematic and microscopy image showing orthogonal blue and UV light‐activatable CFE system each regulating the expression of one subunit of β‐Gal enzyme within synthetic cells. Enzyme activity (shown in yellow) was only observed in regions where UV and blue light patterns intersect. The scale bar is 400 µm. The figure is adapted with permission from ref [63], 2023 the authors CC‐BY 4.0.

One approach to light controlled CFE involves the incorporation of photocleavable groups into DNA to initially block transcription. In one example, a minimal *Escherichia coli* S30 extract and plasmid DNA caged with photolabile protecting groups were encapsulated within nanoscale synthetic cells. Upon UV irradiation (365 nm), the photocage was removed, initiating protein synthesis in vitro and in mice [60]. Alternatively, UV light‐activated DNA (uvLA‐DNA) templates have been constructed by modifying multiple thymines in the promoter with UV‐light cleavable biotin groups that bind multiple streptavidin molecules, efficiently blocking transcription by T7 polymerase. Exposure to UV light cleaved these groups, allowing T7 polymerase to transcribe the downstream genes [61]. These uvLA‐DNA templates were then used to spatially pattern the production of the membrane protein pore, ɑ‐hemolysin (ɑHL), in 3D‐printed synthetic cell tissues, enabling selective exchange of small molecules and electrical signal transmission [61]. The same CFE system has also been applied to photoregulate the synthesis of bacterial quorum sensing molecules, as described in the later communication section [62]. Moreover, a similar strategy employing a different photocleavable‐linker led to the development of a blue light‐activatable DNA (bLA‐DNA) (455 nm activation). Combining uvLA‐DNA and bLA‐DNA within synthetic cells enabled the construction of a dual‐wavelength light‐controlled CFE AND‐gate with each light channel controlling the production of one part of a split β‐galactosidase (β‐Gal) enzyme. Here, only local co‐activation with both wavelengths triggered functional β‐Gal expression within the synthetic cells [63] (Figure 4).

Alternatively, optogenetic tools have been integrated into CFE systems in synthetic cells to achieve light‐regulated gene expression. One possibility involved supplementing the CFE with the blue light‐switchable DNA‐binding protein EL222, which activates downstream gene expression from a blue light‐inducible promoter [64]. This strategy has been employed in synthetic cells to induce the expression of the reporter monomeric red fluorescent protein (mRFP1) under blue light [65]. In addition, the blue light‐regulated two‐component system YF1/FixJ has been directly incorporated into an *E. coli* lysate‐based CFE system, allowing both activation and repression of gene expression under blue light, depending on the design of the genetic circuit [66].

### 2.4 Communication

Cells have the ability to communicate a variety of physiochemical changes to other cells through chemical signals that operate across cellular and organismal length scales [67]. In cellular communication, the timing and spatial localization of the released signals are critical, as they determine how information is transmitted and perceived. Consequently, external light stimuli have been widely employed to regulate communication within synthetic cell communities [68] and hybrid communities of synthetic and natural cells [62]. Communication can be regulated through light triggering the release of chemical messengers [69] or light spatially organizing sender and receiver cell populations [70]. More recently, synthetic cells capable of generating light as a signal themselves have demonstrated that light‐based communication is feasible at the cellular scale [71].

The most direct strategy to regulate communication involves controlling the release and propagation of chemical signals using light. Beyond the light‐triggered release of membrane impermeable molecules using photoswitchable lipids described previously, light‐responsive rotary molecular motors have been embedded in synthetic organelles. Under light illumination (430 nm), these motors facilitated the release of the small molecule, fluorescein‐di‐β‐D‐galactopyranoside (FDG) from the organelles, initiating inter‐ or intracellular reaction cascades [69]. Meanwhile, in the case of synthetic cells with semipermeable protein‐based membranes, photocleavage of DNA complexes has been employed to release single stranded DNA signals, forming diffusion‐based chemical gradients reminiscent of morphogen gradients observed during embryonic development [72].

Aside from the release of chemical signals, communication has also been regulated by the spatial organization of sender and receiver cells. More precisely, when the chemical message from the sender cell is diffusional and short‐ranged, only nearby receivers can respond. In a community comprising one sender and two potential receivers, orthogonal blue light (iLID and Nano) and red light (PhyB and PIF6) switchable protein pairs were used to mediate adhesions between specific sender‐receiver pairs. Depending on the illumination wavelength, distinct self‐sorting patterns with different distances between the sender and the two receivers emerged, leading to selective proximity‐based communication [70]. In another example, communication between adhered synthetic cells could be induced through the activation of connexin nanopores by illumination with IR or UV light. The light would cause protease cargo release from populations of organelles embedded in the adhered sender and receiver synthetic cells. The protease would enable cleavage of a bulky group attached to the nanopores and thus allow insertion into the synthetic cell membrane and the formation of a connected pore between the sender and receiver cells, facilitating cargo transport [73].

**FIGURE 5:**
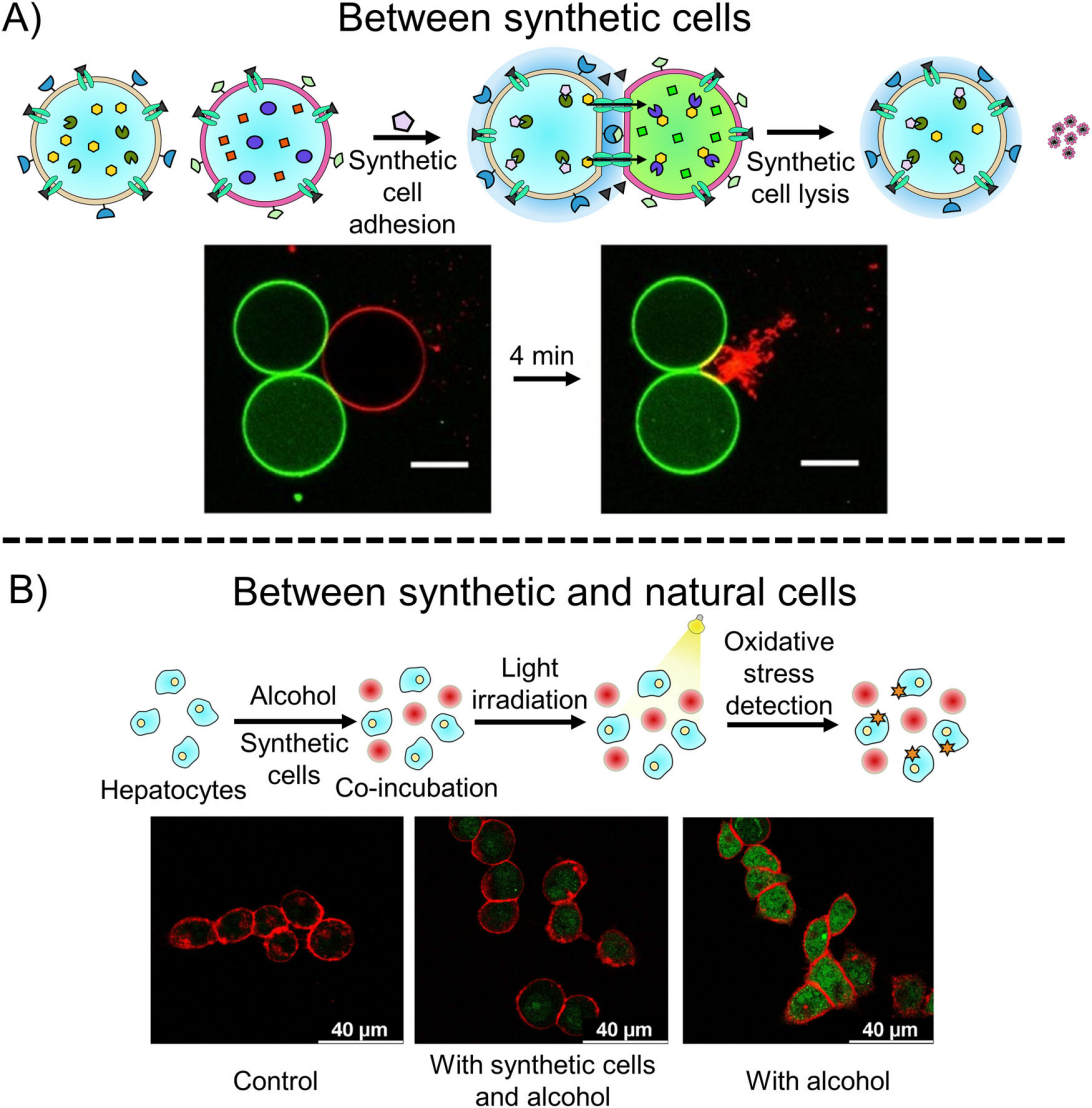
Light‐induced communication in synthetic cells. (A) A diagram showing adhesion between synthetic cells triggered by a bioluminescent signal from the sender cell (shown in green). On adhesion, Ca^2+^ is transferred to the receiver through protein pores present between the synthetic cells, leading to the activation of phospholipase and lysis of the receiver cell population (shown in red). The microscopy images show the selective lysis of the receiver synthetic cell through this communication pathway. Scale bars are 20 µm. (B) Synthetic cells are co‐incubated with hepatocytes and alcohol. On light irradiation, the synthetic cells reduce the amount of alcohol present, thus reducing the oxidative stress of the hepatocytes. The microscopy images show the reduction of oxidative stress (shown in green) in the sample with synthetic cells and alcohol in comparison with the cells only incubated with alcohol. Scale bars are 40 µm. Part (A) is adapted with permission from ref [71], 2021 the authors CC‐BY 4.0. Part (B) is adapted with permission from ref [80], 2025 the authors CC‐BY 4.0.

Starting from synthetic cells, light has also been employed as a communication signal itself. In a first example, *Renilla* luciferase was encapsulated within synthetic sender cells, so that upon addition of its substrate coelenterazine, a blue bioluminescent signal was generated [71]. This light emission from the sender triggered adhesions to the receiver in response, which are based on the blue‐light‐responsive protein‐protein interaction iLID and Nano [74]. This concept was extended further to a predator‐prey system, in which glowing predator cells adhered to prey cells and transferred calcium ions through α‐HL pores upon adhesion. The influx of Ca^2+^ activated phospholipase activity in the prey, leading to its membrane degradation and lysis [71] (Figure 5A). In a different approach, bioluminescence generated at adhesion sites between synthetic sender and receiver cells induced light‐driven membrane localization of the fusion protein SspB‐mCherry [75]. Alternatively, chemiluminescent signaling was achieved through the reaction of luminol and H_2_O_2_ inside the synthetic sender, enabling localized protein recruitment [76] and self‐regulated and bidirectional communication between the adhered synthetic cells [77].

Similar light‐regulated strategies have been extended to communication between synthetic and living cells, including fungal, bacterial, and mammalian cells [62, 65, 78, 79, 80, 81]. Communication with bacteria has been achieved using lipid vesicles incorporating UV‐responsive lipids, which released inducer molecules upon irradiation to activate gene expression in the surrounding bacteria [78]. Alternatively, synthetic cells equipped with light activated DNA and a CFE system, produced the quorum sensing molecule N‐isovaleryl‐l‐homoserine lactone (IV‐HSL) upon UV irradiation, thereby activating quorum sensing in the surrounding bacteria [62]. In communication with mammalian cells, synthetic cells containing photocatalytic polymer‐based organelles have been used to protect hepatocytes from oxidative stress caused by alcohol exposure. Under blue light illumination, the organelles regenerated NAD^+^, which fueled an enzymatic cascade involving alcohol dehydrogenase (ADH) and aldehyde dehydrogenase (ALDH), which converted ethyl alcohol into nontoxic acetate [80] (Figure 5B). In other studies, light triggered release of siRNA from synthetic cells enabled targeted gene silencing in mammalian cells [81] and light activatable DNA aptamers on synthetic cells activated the MET‐AKT‐ERK signaling axis [79]. Finally, synthetic cells capable of producing light have been shown to communicate with light sensitive fungal cells, demonstrating that light‐based communication is also possible in hybrid communities [65].

### 2.5 Growth, Shape Changes, and Division

Living cells can change their shapes, grow, and divide, enabling them to proliferate. During these processes, the change in surface‐to‐volume ratio is dynamically modulated by membrane associated processes such as lipid synthesis and membrane trafficking [82], as well as by intracellular and extracellular factors including cytoskeletal dynamics, mechanical stress, and osmotic changes [83]. Many of these aspects have now been regulated with light within synthetic cells using a variety of different photoactive molecules [84].

One of the most common photoregulation strategies to alter synthetic cell membranes, often accompanied by shape changes, is through the incorporation of lipids bearing photoswitchable tail groups. Lipid analogues with photoswitchable azobenzene (AB) tails have been used to grow giant vesicles and synthetic tissues from lipid aggregates under UV illumination [22, 85]. Similarly, the spiking of other light‐responsive small molecules into lipid mixtures has enabled the photoinduced formation of lipid vesicles under UV light [86]. Incorporation of AB‐containing lipids or amphiphiles into vesicles also provides a handle to manipulate shape transformations, including increases in membrane area, reversible budding [87], tubulation [88], pearling of membrane tubes [89], and domain formation [90]. Such light‐triggered deformations in vesicles can even be translated into collective contractions of prototissues [91]. While most studies rely on UV or blue light, vesicles of synthetic lipids containing tetra‐ortho‐chlorinated AB groups can undergo shape changes and budding under red light illumination [92]. More recently, the incorporation of molecular machines, including light‐driven molecular motors [93] and photoresponsive rotaxanes [94], have enabled deformation of lipid‐based synthetic cells. Moreover, photoswitchable lipids have been employed to regulate the interactions and endocytosis of biomolecular condensates and lipid‐based synthetic cells [95]. Beyond membrane‐bound systems, membraneless synthetic cells have been formed from photoswitchable coacervates [95, 96] and the light‐directed assembly of DNA condensates containing AB groups within a droplet has enabled the deformation and shaping of the surrounding droplet [26]. Light‐responsive DNA condensates encapsulated with lipid synthetic cells have also been shown to cause membrane wetting and budding on UV illumination [97]. In an alternate approach that uses living cells and does not rely on synthetic lipids or photoresponsive groups, *E. coli* cells expressing a light‐driven inward proton pump were used to generate a light‐triggered change in pH. These pH variations modulated the membrane deformation in adjacent synthetic cells by controlling the binding of pH sensitive DNA origami rafts to their membranes [98]. In comparison to the other examples, this approach uses light‐responsive living cells to alter the behavior of the synthetic cells, instead of using synthetic machinery present in the synthetic cells.

**FIGURE 6:**
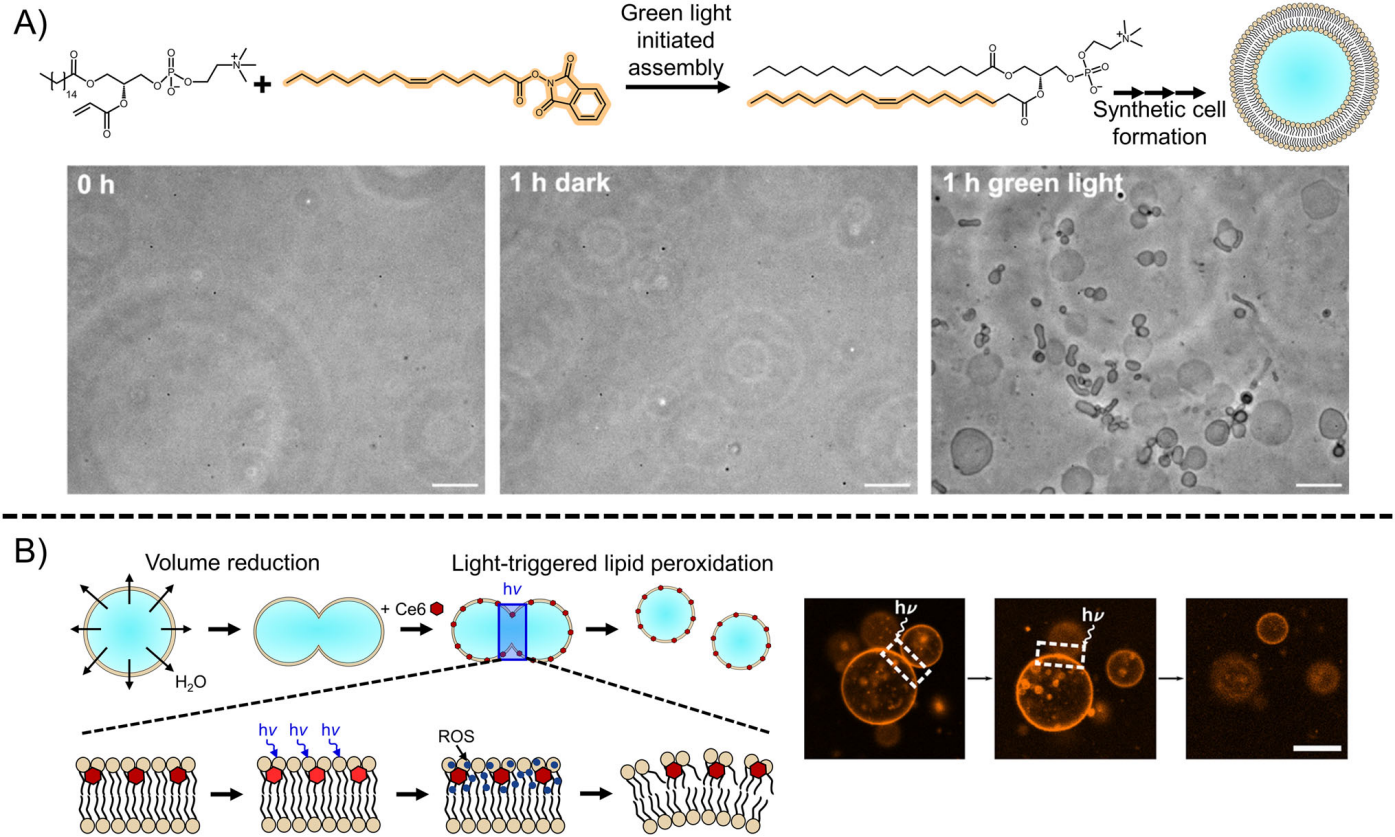
Examples of light‐initiated growth and division in synthetic cells. (A) Green light‐triggered chemical reaction leading to the de novo synthesis of phospholipids results in the formation and growth of lipid vesicles, as microscopically visualized. The scale bars are 20 µm. (B) A diagram with fluorescence microscopy images demonstrating the division of synthetic cells under light. The synthetic cells were deflated and light‐triggered lipid peroxidation was initiated by using the photosensitizer Ce6 embedded in the synthetic cells. The peroxidation led to the division of the synthetic cells, shown in the series of microscopy images. The scale bars are 10 µm. Part (A) is adapted with permission from ref [100], 2025 the authors CC‐BY 4.0. Part (B) is adapted with permission from ref [102], 2021 the authors CC‐BY 4.0.

Achieving growth of synthetic cells through the incorporation of new membrane building blocks rather than only membrane expansion and powering this process with light remains far more challenging. To enable light‐dependent fusion, gold nanoparticles were attached to the membrane of lipid vesicles, which were dragged together using optical traps. Upon contact, the vesicles fused together due to the large amount of local heat generated from the irradiation of the attached gold nanoparticles [99]. In a remarkable example, photochemical synthesis of phospholipids enabled de novo vesicle formation and growth [100] (Figure 6A). In this example, activated fatty acids reacted with secondary precursors to yield a range of biologically relevant lipids via a novel, biocompatible photoredox lipid ligation chemistry. In a step away from phospholipid membranes, fatty acid derived monomers with strained cyclic disulfide groups generated highly reactive thiyl radicals upon light activation, leading to oligomerized fatty acid membrane structures and condensates with diverse morphologies [101].

Division represents the most extreme form of membrane deformation. While photoswitchable membrane molecules can induce bud formation, achieving neck fission and symmetric division of synthetic cells into two daughter compartments has proven more difficult. To address this, the photosensitizer Chlorin e6 (Ce6) was incorporated into lipid membranes to induce local lipid peroxidation with illumination. Membrane peroxidation resulted in pore formation, changes in the spontaneous curvature of the membrane, neck fission, and ultimately the division of lipid‐based synthetic cells [102] (Figure 6B). In another strategy, division was achieved through osmotic changes in the environment of phase separated GUVs. These synthetic cells were placed in a solution containing a photocleavable molecule that, upon illumination, split into three smaller fragments, increasing the osmolarity of the surrounding medium. This triggered water efflux from the vesicles, increased the surface‐to‐volume ratio, and ultimately induced light‐driven budding and symmetric division [103].

### 2.6 Motility

Motility is another aspect sought in synthetic cells, as it underlies a wide range of biological processes, including bacterial taxis [104], immune response [105], and wound healing [106]. Reflecting the diverse strategies that living systems have evolved to achieve motility [107], light has been employed in synthetic cells to generate movement in various ways by modulating adhesions [108], producing forces through nanofiber formation [109], which mimics motility driven by actin assembly and treadmilling [110], repeated contractions of flagellar leading to the generation of asymmetric forces [111], and thermal gradients [112].

One strategy for cell motility is mesenchymal or adhesion‐based migration, where new adhesions form at the leading edge while detachment occurs at the trailing edge. To mimic this dynamic and asymmetric adhesion behavior, photoswitchable adhesions were introduced. Lipid‐based synthetic cells adhered to a functionalized glass substrate under blue light and detached in the dark. By selectively illuminating only parts of the synthetic cell, it only formed adhesions at the front, and its migration was guided into illuminated regions of the glass substrate [108]. This strategy was later adapted to enable motility on fluid and more biomimetic supported lipid bilayers to establish how receptor and ligand mobility and clustering impact motility [113].

**FIGURE 7:**
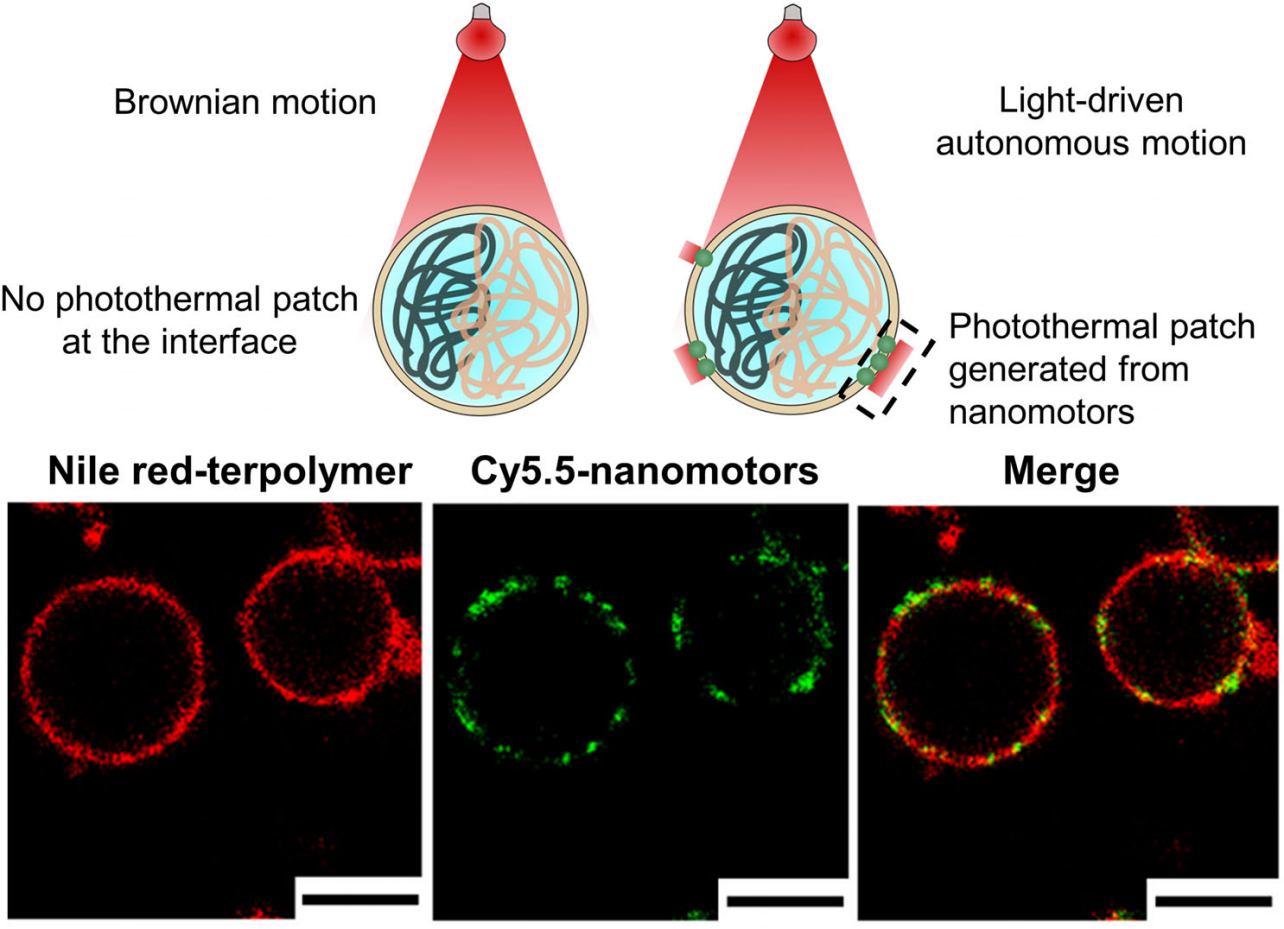
Light‐directed motility within synthetic cells. An illustration with associated microscopy images showing the formation of synthetic cells with near infrared‐light‐responsive nanomotors. The nanomotors were adhered to a terpolymer membrane on the outside of the synthetic cells. Upon irradiation, motion of the synthetic cells was initiated. The scale bars are 5 µm. The figure is adapted with permission from ref [114], 2025 the authors CC‐BY 4.0.

Alternatively, adhesion independent motility has been achieved through asymmetrically distributed reactions in synthetic cells, which generate propulsion forces. For instance, terpolymer stabilized coacervate‐based synthetic cells were coated in a patchy fashion with nanomotors containing gold nanoparticles. Upon near infrared light illumination, the nanoparticles converted light into heat, generating a temperature gradient across the synthetic cell that drove directional motion. The density of nanomotors on the surface controlled the magnitude of movement, allowing tunable light‐induced motility [114] (Figure 7). Other examples include the use of molybdenum disulfide (MoS_2_) nanoparticles, which produce protons upon red light irradiation. These protons fueled ATP synthesis through ATP synthase, induced localized electroosmotic flow, and movement of negatively charged MoS_2_ particles inside the synthetic cells, propelling the system towards the light source. This propulsion mechanism was then demonstrated in a biological context, where the synthetic cells migrated towards a target site within mice for localized delivery [112]. Alternatively, DNA‐based synthetic cells have exploited AB photoswitches to transition regions of the synthetic cells between gel, liquid, and dissociated states. By locally cycling between these state transitions using UV light, a force could be generated, which propelled the synthetic cell in a targeted direction [115]. In another example, controlled light‐driven assembly of AB‐containing DNA condensates within a droplet has been able to mimic cell‐crawling behavior [26].

## 3 Photoswitchable Toolkit for Next‐Generation Synthetic Cells

The sections above highlight how light can energize and regulate a wide range of cell‐like functions within synthetic cells. These functionalities are summarized below (Table 1), along with the wavelength of light used and type of light‐responsive component. A broad spectrum of components from synthetic small molecules, photocatalysts and nanoparticles to light‐responsive proteins and organelles/bacteria can drive biological functionality in synthetic cells. These components differ in wavelength of response, reversibility, and size, which often dictate their application. Despite this extensive toolkit, several challenges remain for advancing the integration of light responsiveness in synthetic cells and several design concepts can be deduced.

**TABLE 1:** Synthetic cell functionalities that utilize light.

Light	Function	Light‐responsive component
250–400 nm (UV)	Compartmentalization [21, 22, 24, 25, 26, 28, 34, 35], Protein synthesis [60, 61, 62, 63], Communication [62, 73, 78, 81], Growth, shape changes, and division [22, 26, 85, 86, 87, 88, 89, 90, 91, 94, 95, 96, 97, 101], Motility [26, 109, 115]	Photoswitchable molecule [22, 25, 26, 85, 86, 87, 88, 89, 90, 91, 94, 95, 96, 115], Photopolymerizable lipid [21, 34, 35, 73, 78], Photocleavable group [24, 28, 60, 61, 62, 63, 81, 97, 109], Photopolymerizable monomer [101]
400–450 nm (Violet)	Compartmentalization [27, 32, 36], Communication [69, 72], Growth, shape changes, and division [93, 102, 103], Motility [115]	Photocleavable group [27, 36, 72, 103], Light‐responsive protein [32], Photoswitchable molecule [69, 115], Molecular motor [93], Photosensitizer [102]
450–500 nm (Blue)	Compartmentalization [23, 29, 31, 33, 37, 80], Energy supply and metabolism [42, 51, 54], Protein synthesis [63, 65], Communication [65, 70, 71, 74, 75, 76, 77, 80], Growth, shape changes, and division [85, 87, 88, 89, 92, 94, 95, 96], Motility [108, 113]	Light‐responsive protein [23, 29, 31, 33, 42, 65, 70, 71, 74, 75, 76, 77, 108, 113], Photocatalyst [37, 51, 54, 80], Photocleavable group [63], Photoswitchable molecule [85, 87, 88, 89, 92, 94, 95, 96]
500–570 nm (Green)	Compartmentalization [20], Energy supply and metabolism [46], Growth, shape changes, and division [100], Motility [111]	Nanoparticles [20], Light‐responsive protein [46, 111], Photocatalyst [100]
570–620 nm (Yellow/Orange)	None	None
620–750 nm (Red)	Compartmentalization [30, 33], Energy supply and metabolism [46], Communication [70],Growth, shape changes, and division [92], Motility [112]	Light‐responsive protein [30, 33, 46, 70], Photoswitchable molecule [92], Nanoparticles [112]
> 750 nm (Near infrared/infrared)	Energy supply and metabolism [47], Communication [73, 79], Growth, shape changes, and division [99], Motility [114]	Photosynthetic organelle [47], Nanoparticles [73, 79, 99, 114]
White light (No defined wavelength)	Energy supply and metabolism [11, 40, 41, 43, 44, 45, 48, 49, 50, 52, 53], Growth, shape changes, and division [98]	Thylakoid membrane [11, 49], Light‐responsive protein [40, 41, 43, 44, 45], Nanoparticles [43, 50], Cyanobacteria [48], Photocatalyst [52, 53], *E. coli* [98]

Firstly, the majority of light responses in synthetic cells still use UV to blue light and the number of examples using wavelengths in the red are few. Low wavelength light has limited penetration in biological tissues and can cause photodamage compared with longer wavelengths [116], restricting the in vivo and in cellulo applicability of low wavelength‐responsive systems. Photocleavable and photoswitchable small molecules are often used in conjunction with <450 nm light due to the energy needed to alter chemical bonds [117], while light‐responsive proteins and cellular components generally operate with visible light of >450 nm. The development of visible light‐switchable molecules therefore remains an active research area [16]. Metal nanoparticles, which convert light to heat and absorb mostly red light, are promising for biological applications [112], though local heating can cause unspecific effects. Visible light photocatalysis [52] offers yet another strategy to circumvent issues associated with low wavelength photoactivation. Another challenge is that using light as an external trigger requires optical transparency at the activation wavelength, which again limits deep tissue applications. Recent work shows that bioluminescent [65, 71] and chemiluminescent [77] blue light produced inside synthetic cells is able to activate blue‐light‐responsive proteins, suggesting a way around this limitation. Through the incorporation of higher wavelength‐responsive photoswitches [118] and proteins [119], and luminescent activation strategies, light‐responsive synthetic cells can become more compatible with biologically relevant environments and in vivo applications.

Secondly, consideration needs to be given to the size and reversibility of the light‐responsive element, depending on the targeted function. For processes requiring membrane remodeling, such as compartmentalization, growth, division or shape changes, photoswitchable lipid analogues are ideal for membrane incorporation. In contrast, metabolism and energy conversion are better driven by photocatalytic proteins and light‐driven ion channels, sometimes replaced by synthetic photocatalysts. When life‐like processes rely primarily on proteins, simply integrating light‐responsive proteins or photoswitchable small molecule modulators can be the most straightforward strategy. For photoregulating life‐like processes, dynamics are a central consideration in the choice of the light‐responsive components. Irreversible changes, triggered by a single light pulse with photocleavable groups or photoinduced reactions, can initiate downstream steps and are useful for processes like division, CFE activation or cargo release. Reversible photoswitches, including photoisomerizing small molecules and photoswitchable proteins, enable dynamic temporal control relevant in processes like shape changes, migration, communication, and metabolic regulation.

Current examples often only utilize a single wavelength of light, and often do not take advantage of the micrometer spatial control that light can offer. Incorporating various components responsive to different wavelengths of light allows the inclusion of multistep light dependent reactions [120], light‐based logic gates for biocomputation [121, 122], or spatial patterning with different colors [63]. One possibility is the incorporation of several channelrhodopsins, which are widely used in optogenetics, into synthetic cell membranes, allowing the influx and efflux of different ions to be modulated by distinct wavelengths [123, 124]. Spatiotemporal control of ion transport could then be used to regulate a variety of synthetic cell behaviors. These concepts would also expand the design space of light‐responsive materials within synthetic cells, allowing functionality to be tuned by wavelength sequence, intensity, irradiation time, or spatial targeting. However, utilizing multiple light‐activated components limits the range of wavelengths available for fluorescence imaging, which is the predominant method to analyze synthetic cell behavior. To overcome this, alternative imaging methods, which work in a different part of the electromagnetic spectrum, such as stimulated Raman scattering microscopy [125] or radioluminescence microscopy [126], along with non‐optical methods including biomolecule detection through nanopores [127] or mass spectroscopy [128] could be used. There is also an opportunity to use other types of waves, most notably ultrasound, electric and magnetic waves, which have been used to pattern and enable directed motion in synthetic cell communities [129, 130, 131, 132, 133], opening orthogonal avenues for systematically controlling and investigating bioinspired functionality in tandem with light.

Finally, the complexity of light‐responsive synthetic cells could be increased by following a cellular bionics approach [134] and incorporating organisms or organelles that generate or respond to light, such as bioluminescent bacteria [135] and optogenetically engineered mammalian cells [136]. These biohybrid platforms could drastically increase functional complexity without requiring large numbers of encapsulated biomolecules. Furthermore, the synthetic cell can serve as a protective chassis [137], allowing the light‐responsive biological organisms to operate across a wider range of environments [138], increasing the range of potential applications.

In conclusion, remarkable progress has been made using light to recreate biological form, function, and behavior in synthetic cells. Yet significant opportunities remain to further incorporate light responsivity to advance synthetic cells towards replicating living cells. As outlined above, future directions could involve increasing the utilization of higher wavelength light‐responsive components, the integration of multiple spatiotemporally‐controlled processes in one system and the construction of biohybrid systems. The formation of these next‐generation light‐responsive synthetic cells and tissues could also be aided through manufacturing methods such as droplet microfluidics [139] and 3D printing [140], which will offer new opportunities to spatiotemporally control both the manufacturing and activation of functionality within the synthetic cells and tissues. Moreover, with these processes, machine learning tools could be integrated [141] to comprehensively probe and evaluate the light dependent behavior, enabling a deeper understanding of the interactions within light controlled synthetic cells. Achieving this will require continued cross‐disciplinary integration of light responsive systems from biology, chemistry, and material science. At the same time, these efforts will unlock a range of emergent functionalities and help realize the potential of light‐responsive synthetic cells in biotechnology.

## Author Contributions

**Matthew E. Allen**: conceptualization, writing – original draft, writing – review and editing, supervision. **Saskia Frank**: writing – original draft. **Mehaiarii Louis**: writing – original draft. **Atreyee Saha**: writing – original draft. **Ali Heidari**: conceptualization, writing – review and editing. **Seraphine V. Wegner**: conceptualization, writing – review and editing, supervision, funding acquisition.

## Conflicts of Interest

The authors declare no conflicts of interest.

## Data Availability

Data sharing is not applicable to this article as no datasets were generated or analyzed during the current study.
